# Psychopathy facets and suicide risk among violently injured adults

**DOI:** 10.3389/fpsyt.2026.1850812

**Published:** 2026-06-22

**Authors:** Nicholas D. Thomson, Brooklyn A. Worthen, Jessica J. James

**Affiliations:** 1 Department of Surgery, Virginia Commonwealth University, Richmond, VA, United States; 2 Departments of Psychology and Psychiatry, Virginia Commonwealth University, Richmond, VA, United States

**Keywords:** externalizing, psychopathy, suicidal behavior, suicide risk, violent injury

## Abstract

**Background:**

Suicide risk remains difficult to predict, particularly among populations exposed to interpersonal violence. Although psychopathic traits have been linked to suicidal behavior, emerging evidence suggests these associations vary across psychopathy domains. The present study examined whether overall psychopathy and specific psychopathy facets were associated with suicide risk among violently injured adults.

**Methods:**

Participants were 458 violently injured adults (72% male; *M_age_* = 32.76 years) recruited from an urban trauma center in the United States. Participants completed measures assessing psychopathic traits, suicide risk, post-traumatic stress disorder (PTSD), and substance use. Hierarchical regression analyses were conducted to examine associations between psychopathy and suicide risk while controlling for age, sex, race, PTSD and substance use.

**Results:**

After controlling for age, sex, race, PTSD, and substance use, total psychopathy was positively associated with suicide risk. When psychopathy facets were examined, only the Lifestyle facet emerged as a significant independent predictor of suicide risk. Sex did not moderate associations between psychopathy and suicide risk.

**Conclusions:**

These findings suggest that the association between psychopathy and suicidality in violence-exposed populations may be more strongly related to traits reflecting behavioral dysregulation and impulsivity than to other psychopathy domains. Lifestyle-related psychopathic traits may be associated with greater suicide vulnerability through elevated impulsivity and increased engagement in risk-taking during periods of distress. Results highlight the importance of examining psychopathy as a multidimensional construct and suggest that externalizing personality traits may represent one potential pathway associated with suicide risk among violently injured adults.

## Introduction

Suicide represents a major global public health concern and remains one of the leading causes of death worldwide, with rates continuing to rise over the past decade (1, 2). Despite decades of research, accurately predicting suicidal behavior remains a major challenge. Most models of suicide emphasize affective distress, hopelessness, and perceived burdensomeness as key drivers of suicidal ideation (3, 4). However, these mechanisms do not fully explain why only a subset of individuals who experience suicidal thoughts ultimately engage in suicidal behavior. Increasingly, researchers have emphasized the role of behavioral dysregulation and impulsivity in facilitating the transition from suicidal ideation to action (5, 6).

Psychopathy represents a personality construct that may be relevant for understanding behavioral risk processes. Psychopathy is a multidimensional personality construct consisting of separable domains that capture distinct patterns of personality and behavioral traits, including affective detachment, interpersonal manipulation, impulsive lifestyle, and antisocial behavior (7, 8). These domains differ in their cognitive, emotional, and behavioral correlates (7). Behavioral and lifestyle traits have been associated with impulsivity, sensation seeking, and broader patterns of behavioral dysregulation, whereas affective and interpersonal traits reflect emotional shallowness and manipulative interpersonal style (8, 9).

Emerging evidence suggests that psychopathic traits may be associated with suicidal thoughts and behaviors, although these associations appear to vary across psychopathy domains (10). Meta-analytic evidence suggests that traits reflecting behavioral dysregulation, particularly those captured by the lifestyle and antisocial facets, show the strongest positive associations with these suicide-related outcomes, whereas affective and interpersonal traits show smaller and less consistent associations (10). These findings suggest that behavioral dysregulation, rather than emotional detachment, may represent the psychopathic feature most relevant to suicide risk.

The distinction between psychopathy facets is theoretically meaningful in the context of suicide risk. Impulsivity and behavioral dysregulation are well-established risk factors for suicidal behavior, particularly in contexts of intense affect (5, 6). Individuals high in disinhibitory traits may act quickly in response to distress, fail to consider long-term consequences, and repeatedly engage in painful or provocative experiences that lower fear of harm (11, 12). Historically, psychopathy has been viewed as conferring relatively low risk for suicide, a perspective rooted in early clinical descriptions emphasizing emotional detachment (13). However, research examining psychopathy at the facet level suggests that this apparent protective effect may be driven by interpersonal and affective traits rather than the broader psychopathy construct. For example, empirical studies indicate that suicidal behavior is more strongly associated with Factor two traits, reflecting lifestyle instability and antisocial behavior, whereas Factor one traits, capturing interpersonal manipulation and affective detachment, show weaker or inconsistent associations with suicide (10, 14). Clarifying which domains of psychopathy are most strongly linked to suicide risk may therefore help refine theoretical models and improve clinical risk assessment.

Despite growing evidence linking psychopathy to suicidality (10), relatively little research has examined these associations in violence-exposed community populations. Most prior studies have relied on heterogeneous samples drawn from incarcerated or psychiatric populations (10), leaving uncertainty about whether observed associations generalize beyond settings characterized by elevated psychiatric severity, institutional constraints, and treatment involvement. Violently injured adults represent a population with elevated risk for subsequent violence and suicide (15). They share features commonly observed in justice-involved samples, including exposure to interpersonal violence and elevated behavioral dysregulation, yet they remain community-dwelling and unconstrained by institutional environments. Examining psychopathy–suicide associations in this population addresses an important gap in the literature because incarcerated and psychiatric samples are often characterized by factors that may influence both psychopathic traits and suicide risk. Investigating these associations in a violently injured population, therefore provides an opportunity to explore whether previously observed associations generalize to a high-risk community population and whether specific psychopathy domains demonstrate unique associations with suicide risk outside correctional or psychiatric settings.

The present study examined whether psychopathy is associated with suicide risk among violently injured adults. First, we tested whether overall psychopathy was positively associated with suicide risk, controlling for post-traumatic stress disorder (PTSD) and substance use. Second, we examined psychopathy at the facet level to determine whether lifestyle and disinhibition-related traits uniquely predicted suicide risk when affective, interpersonal, and antisocial traits were considered simultaneously. Third, we evaluated whether sex moderated these associations. Based on existing theory and meta-analytic evidence, we hypothesized that overall psychopathy would be positively associated with suicide risk and that lifestyle-related traits would emerge as the strongest independent predictor. Because evidence regarding sex differences in these associations is limited, sex moderation was explored.

## Materials and method

### Participants

This study included 458 violently injured adults, primarily male (72%), aged 18 to 75 years (*M* = 32.76, *SD* = 12.79). Most participants self-identified as Black/African American (76%), followed by White/Caucasian (16%), Asian (0.6%), American Indian (0.4%), or other (5.1%), with 76% identifying as non-Hispanic. Regarding injuries leading to hospitalization, 53% had sustained gunshot wounds, 8% stab wounds, and 39% assault wounds, while 22% reported prior hospitalization for violence-related injuries. Regarding education and employment, 88% had completed 12^th^-grade education or earned a GED, and 29% were unemployed and looking for work. The present study had a recruitment rate of 82%.

### Procedure

Participants were recruited from an urban Level 1 Trauma Center in Virginia through either the emergency department or inpatient services as part of a broader study examining retaliatory gun violence (R01CE003296, PI: Thomson). Individuals were eligible if they were over the age of 18 years, had experienced a violence-related injury, and were not incarcerated. The present study focuses specifically on violently injured individuals, a population known to be at elevated risk for subsequent violence and violence-related re-injury. Prior to providing consent, participants were informed that the study was conducted solely for research purposes and that participation would not affect their medical treatment. Participants completed the Self-Report Psychopathy Scale at baseline and received $160 in compensation for their involvement in the larger study. All study procedures were approved by the Virginia Commonwealth University Institutional Review Board, and the project was covered by a Certificate of Confidentiality issued by the Centers for Disease Control and Prevention.

### Measures

#### Psychopathy

Psychopathy was assessed using the Self Report Psychopathy Scale - Short Form (16), a 29-item measure designed to capture four core facets of psychopathy. These facets include Interpersonal traits (7 items; for example, “I have pretended to be someone else in order to get something”), Affective traits (7 items; for example, “I don’t feel bad about hurting people”), Lifestyle traits (7 items; for example, “I’ve often done something dangerous just for the thrill of it”), and Antisocial traits (7 items; for example, “I have tricked someone into giving me money”). Participants responded to items using a 5-point Likert scale ranging from 1 (disagree strongly) to 5 (agree strongly). Scores were averaged such that higher values reflected greater levels of psychopathic traits. In the current sample, the SRP-SF demonstrated strong internal consistency overall and across facets (total scale α = .92; Interpersonal α = .82; Affective α = .78; Lifestyle α = .82; Antisocial α = .79).

#### Suicide risk

Suicide risk was assessed using the Suicidal Behaviors Questionnaire - Revised (17), a brief 4-item self-report measure designed to index clinically relevant suicidal thoughts and behaviors across multiple domains. The SBQ-R assesses (1) lifetime suicidal ideation and attempt history, (2) frequency of suicidal ideation in the past 12 months, (3) threat of suicide attempt, and (4) perceived likelihood of future suicidal behavior. Items are summed to yield a total risk score, with higher scores reflecting greater suicide risk. The SBQ-R showed good internal consistency in the present sample (α = .86).

#### Post-traumatic stress disorder

PTSD was assessed using The Post-Traumatic Stress Disorder Checklist DSM-5 (PCL-5) (18). The PCL-5 is a 20-item self-report measure that assesses the 20 DSM-5 symptoms of PTSD. Items are scored on a 5-point Likert scale ranging from 0 (Not at all) to 4 (Extremely). Consistent with prior research, a dichotomous PTSD variable was created using the recommended clinical cutoff score (≥ 31), with higher scores indicating clinically significant PTSD symptoms. The PCL-5 demonstrated excellent internal consistency in the present sample (α = .94).

#### Substance use

Substance use was assessed using the Drug Abuse Screening Test (DAST-10) (19). The DAST-10 is a 28-item self-report measure designed to screen for substance use in the past 12 months. Items are scored dichotomously (Yes/No), with higher scores reflecting greater substance-related problems. For the present study, a dichotomous substance use variable was used to indicate the presence or absence of clinically significant substance use problems. The DAST-10 demonstrated good internal consistency in the current sample (α = .83).

### Analytic plan

First, zero-order correlations were examined using Spearman correlations for binary variables and Pearson correlations for continuous variables. The coding approach for race (0 = other, 1 = White) was used to broadly account for social and structural factors that may influence health outcomes differently across racial groups and is consistent with prior research that uses race as a covariate adjustment variable. Next, we conducted two hierarchical regressions testing if total scores and facet scores were related to suicide risk and if sex moderated this association. Total psychopathy scores and psychopathy facet scores were standardized (z-scored) before regression analyses. All analyses were performed in R (20). *A priori* sensitivity analyses were conducted for hierarchical multiple regression models evaluating incremental variance explained (ΔR²), assuming α = .05 and 80% power. In the total-psychopathy moderation model, Step 1 included age, sex, race, PTSD, substance use, and total psychopathy, and Step 2 added the sex × total psychopathy interaction term. With *N* = 458 and seven predictors in the full model, the study was powered to detect an individual interaction effect of approximately *f*² = .017 or larger, corresponding to Δ*R*² ≈.014 given the observed full-model *R*² of .181. In the facet-level moderation model, Step 1 included age, sex, race, PTSD, substance use, and the four psychopathy facets, and Step 2 added the four sex × facet interaction terms. With *N* = 458 and 13 predictors in the full model, the study was powered to detect a Step 2 interaction block effect of approximately *f*² = .027 or larger, corresponding to Δ*R*² ≈.022 given the observed full-model *R*² of .181. Thus, the present study was adequately powered to detect small interaction effects in the total psychopathy model and small-to-moderate interaction effects in the facet-level model. Multicollinearity was evaluated using variance inflation factors (VIFs), which ranged from 1.04 to 4.98 (tolerances .20 to .96), indicating acceptable multicollinearity.

## Results

### Correlations between primary study variables

Descriptive statistics and zero-order correlations among primary study variables are presented in **Table 1**. Total psychopathy scores were highly correlated with all SRP-SF facets (*r*’s = .84–.89, *p*’s <.001). The psychopathy facets were also moderately to strongly intercorrelated with one another (*r’*s = .63–.71, *p*’s <.001). Suicide risk was positively associated with total psychopathy (*r* = .21, *p* <.001) and with each psychopathy facet, including Interpersonal (*r* = .16, *p* <.001), Affective (*r* = .18, *p* <.01), Lifestyle (*r* = .28, *p* <.001), and Antisocial traits (*r* = .12, *p* <.05). PTSD was positively associated with suicide risk (*r* = .31, *p* <.001), total psychopathy (*r* = .21, *p* <.001), and all psychopathy facets (*r*’s = .16 –.23, *p*’s <.01). Substance use was positively associated with suicide risk (*r* = .21, *p* <.001), total psychopathy (*r* = .38, *p* <.001), and all psychopathy facets (*r*’s = .29 –.38, *p*’s <.001). Age was not significantly related to suicide risk (*r* = .06, *p* = .17) or to total psychopathy (*r* = −.03, *p* = .49). Sex and race were also associated with suicide risk. Specifically, females demonstrated higher suicide risk (*r* = .14, *p* = .003), and White participants also reported higher suicide risk (*r* = .22, *p* <.001).

**Table 1:** Correlations and descriptive statistics among primary study variables.

Measure	*M*	*SD*	1	2	3	4	5	6	7	8	9	10	11
1. Sex	0.28	0.45	–										
2. Race	0.16	0.37	.02	–									
3. Age	32.79	12.92	-.06	.15**	–								
4. Suicide Risk	4.91	3.11	.14**	.22***	.06	–							
5. Psychopathy	56.86	19.46	-.13**	.01	-.03	.21***	–						
6. Interpersonal	12.09	5.18	-.12*	-.02	-.01	.16***	.86***	–					
7. Affective	15.17	5.83	-.09	-.07	-.09*	.18**	.88***	.68***	–				
8. Lifestyle	14.21	5.84	-.09*	.11*	-.04	.28***	.89***	.71***	.71***	–			
9. Antisocial	15.41	5.40	-.13**	.02	.05	.12*	.84***	.66***	.63***	.66***	–		
10. PTSD	30.84	20.03	.22***	.11*	-.03	.31***	.21***	.16**	.17**	.23***	.16***	–	
11. Substance use	1.89	2.38	-.19	.09*	.13*	.21***	.38***	.31***	.29***	.38***	.37***	.12***	–

p <.05*, p <.01**, p <.001***. PTSD, posttraumatic stress disorder.

Pearson correlations were used for associations among continuous variables. Spearman correlations were used for binary variables (sex, race, PTSD, Substance use).

### Total psychopathy scores and suicide risk

Regression results are presented in **Table 2**. In Step 1, age, sex, race, PTSD, substance use, and total psychopathy scores were entered to predict suicide risk in the past 12 months. This model was significant, *F*(6, 439) = 19.28, *p* <.001, explaining 21% of the variance in suicide risk (*R*² = .21). Total psychopathy was a significant positive predictor of suicide risk (*β* = .19, *p* <.001), such that higher psychopathy scores were associated with greater suicide risk even after controlling for PTSD and substance use. Sex and race also emerged as significant predictors, with higher suicide risk observed among females (*β* = .14, *p* <.01) and White participants (*β* = .17, *p* <.001). Clinically significant PTSD levels were also independently associated with greater suicide risk (*β* = .23, *p* <.001).

**Table 2:** Psychopathy and suicide risk.

	Suicide Risk					
	*b*	*SE*	*β*	95% CI	*R^2^*	Δ*R²*
**Step 1**					.21	*F*(6,439) = 19.28, *p* < .001
Age	0.01	0.01	0.02	-0.02, 0.03		
Sex	0.96	0.31	0.14**	0.37, 1.56		
Race	1.43	0.36	0.17***	0.73, 2.13		
PTSD	1.39	0.28	0.23***	0.84, 1.93		
Substance use	0.12	0.06	0.09	-0.01, 0.24		
Psychopathy	0.61	0.15	0.19***	0.31, 0.91		
**Step 2**					.21	*F*(7, 438) = 16.59, p < .001
Age	0.01	0.01	0.02	-0.02, 0.03		
Sex	0.98	0.31	0.15**	0.38, 1.58		
Race	1.41	0.36	0.17***	0.71, 2.11		
PTSD	1.39	0.28	0.23***	0.84, 1.94		
Substance use	0.12	0.06	0.09	-0.01, 0.24		
Psychopathy	0.55	0.17	0.19***	0.21, 0.88		
Psychopathy*Sex	0.21	0.29	0.03	-0.37, 0.79		

p <.05*, p <.01**, p <.001***. PTSD, posttraumatic stress disorder.

In Step 2, the interaction between sex and total psychopathy was added to the model. This interaction did not meaningfully improve model fit, Δ*R*² = .00, Δ*F*(1, 438) = 0.52, *p* = .47, and the interaction term was not significant (*β* = .03, *p* = .47), indicating that sex differences in the association between psychopathy and suicide risk were not detected in the present sample. However, these findings should be interpreted cautiously, given the predominantly male composition of the sample.

### Psychopathy facets and suicide risk

Regression results examining psychopathy facets are presented in **Table 3**. In Step 1, age, sex, race, PTSD, substance use, and the four psychopathy facets (Interpersonal, Affective, Lifestyle, and Antisocial) were entered to predict suicide risk in the past 12 months. This model was significant, *F*(9, 436) = 13.44, *p* <.001, accounting for 22% of the variance in suicide risk (*R*² = .22). Within this model, the Lifestyle facet of psychopathy emerged as a significant positive predictor of suicide risk (*β* = .16, *p* <.05), suggesting that higher lifestyle psychopathic traits were associated with greater suicide risk. Interpersonal, Affective, and Antisocial facets were not significant predictors (*p’s* >.21). Although Antisocial traits demonstrated a positive bivariate association with suicide risk, the direction of the regression coefficient became negative when entered simultaneously with the remaining psychopathy facets. Given the substantial intercorrelations among psychopathy domains, this pattern may reflect shared variance among facets rather than a meaningful protective effect of antisocial traits. Sex and race remained significant predictors, with higher suicide risk observed among females (*β* = .14, *p* <.01) and White participants (*β* = .16, *p* <.001). Clinically significant PTSD symptomology was also independently associated with greater suicide risk (*β* = .22, *p* <.001). Age and substance use were not significantly associated with suicide risk (*p*’s >.07).

**Table 3:** Psychopathy facets and suicide risk.

	Suicide Risk					
	*b*	*SE*	*β*	95% CI	*R²*	Δ*R²*
**Step 1**					.22	*F*(9, 436) = 13.44, p < .001
Age	0.01	0.01	0.03	-0.01, 0.03		
Sex	0.96	0.31	0.14**	0.36, 1.56		
Race	1.34	0.37	0.16***	0.62, 2.06		
PTSD	1.36	0.28	0.22***	0.82, 1.91		
Substance use	0.12	0.06	0.09	-0.01, 0.24		
Interpersonal	0.13	0.207	0.04	-0.28, 0.53		
Affective	0.26	0.21	0.08	-0.15, 0.67		
Lifestyle	0.51	0.22	0.16*	0.06, 0.94		
Antisocial	-0.21	0.22	-0.07	-0.61, 0.19		
**Step 2**					.22	*F*(13, 432) = 9.51, p < .001
Age	0.01	0.01	0.03	-0.01, 0.03		
Sex	0.98	0.31	0.15**	0.38, 1.58		
Race	1.31	0.37	0.16***	0.58, 2.04		
PTSD	1.34	0.28	0.22***	0.79, 1.89		
Substance use	0.11	0.06	0.09	-0.01, 0.24		
Interpersonal	0.18	0.23	0.06	-0.27, 0.64		
Affective	0.15	0.24	0.05	-0.32, 0.62		
Lifestyle	0.42	0.24	0.14	-0.05, 0.92		
Antisocial	-0.14	0.23	-0.05	-0.59, 0.31		
Interpersonal*Sex	-0.41	0.54	-0.07	-1.47, 0.65		
Affective*Sex	0.42	0.47	0.07	-0.51, 1.35		
Lifestyle*Sex	0.56	0.56	0.09	-0.53, 1.66		
Antisocial*Sex	-0.26	0.51	-0.04	-1.25, 0.72		

p <.05*, p <.01**, p <.001***. PTSD, posttraumatic stress disorder.

In Step 2, interaction terms between sex and each psychopathy facet were added to the model. Adding the interaction terms did not significantly improve model fit, Δ*R*² = .01, *F*(4, 432) = 0.73, *p* = .57, and none of the sex by psychopathy facet interaction terms were statistically significant (*p*’s ≥.31), suggesting that sex differences in the associations between psychopathy facets and suicide risk were not detected in the present sample. However, these findings should be considered tentative given the predominantly male composition of the sample.

## Discussion

The present study investigated whether psychopathic traits are associated with suicide risk among violently injured adults and whether specific psychopathy facets differentially predict suicide vulnerability. Consistent with our hypotheses, overall psychopathy was positively associated with suicide risk. However, when psychopathy was examined at the facet level, only the Lifestyle facet emerged as a significant predictor of suicide risk. These findings suggest that the association between psychopathy and suicidality in violence-exposed populations may be driven primarily by traits reflecting behavioral dysregulation and impulsivity rather than by interpersonal manipulation or affective detachment.

The present findings are consistent with theoretical and empirical research linking impulsivity and behavioral dysregulation to suicidal behavior. The Lifestyle facet of psychopathy reflects impulsivity, irresponsibility, sensation seeking, and behavioral instability (8, 21). These characteristics align closely with factors that have been associated with suicidal behavior in prior research (6, 12). Individuals high in impulsive and disinhibited traits may be more likely to act rapidly in response to distress, exhibit reduced consideration of long-term consequences, and engage in risky or painful behaviors that may be associated with reduced fear of self-harm (4, 5, 22). Within violence-exposed populations, repeated exposure to interpersonal harm may also contribute to suicide vulnerability through greater engagement in risky behaviors and exposure to physically painful experiences (15, 23). Thus, individuals characterized by elevated lifestyle-related psychopathic traits may be particularly vulnerable to transitioning from suicidal ideation to suicidal behavior, however future longitudinal research is needed to determine whether these processes function as mechanisms underlying the observed associations.

Importantly, the present findings are consistent with recent meta-analytic evidence indicating that the association between psychopathy and suicidality varies across psychopathy domains (10). Specifically, lifestyle and antisocial traits, reflecting behavioral dysregulation and externalizing tendencies, demonstrate the strongest positive associations with suicidal ideation, suicide attempts, and self-harm, whereas interpersonal and affective traits show weaker and less consistent relationships with suicide-related outcomes (10). The current results further support this distinction by demonstrating that lifestyle-related traits uniquely predicted suicide risk even when other psychopathy facets were considered simultaneously. Although antisocial traits were positively correlated with suicide risk at the bivariate level, they did not uniquely predict suicide risk when other psychopathy facets were considered simultaneously. This pattern may reflect substantial overlap between antisocial behavior and lifestyle impulsivity within psychopathy models. Taken together, these findings underscore the importance of examining psychopathy as a multidimensional construct when evaluating its relationship with suicide risk.

More broadly, the present findings highlight the importance of distinguishing between the interpersonal-affective and lifestyle-antisocial factors of psychopathy. Traditional conceptualizations of psychopathy emphasized emotional detachment, fearlessness, and reduced anxiety, which historically contributed to the view that psychopathy confers relatively low suicide risk (13). However, multidimensional models of psychopathy suggest that the externalizing features of psychopathy, captured by the lifestyle and antisocial facets, may show stronger associations with suicide vulnerability (8, 10). The present findings support this interpretation by suggesting that externalizing traits reflecting impulsivity and behavioral dysregulation may represent one possible explanatory framework for understanding the association between psychopathy and suicide risk, although future longitudinal research is needed to determine the mechanisms underlying these associations.

Sex and race also emerged as significant predictors of suicide risk in the present sample. White participants reported higher suicide risk relative to non-White participants, a finding generally consistent with epidemiological research indicating elevated rates of suicidal ideation, suicide attempts, and suicide mortality among White populations in the United States (24). These differences may reflect broader social, cultural, and healthcare-related factors influencing the expression, reporting, and experience of suicidal thoughts and behaviors rather than characteristics specific to psychopathy. Although females also reported elevated suicide risk in the present sample, this finding should be interpreted cautiously, given the predominantly male composition of the sample.

The present study also extends prior research by examining these associations within a community sample of violently injured adults. Much of the existing literature on psychopathy and suicidality has relied on incarcerated or psychiatric samples (10). Although these populations provide valuable insights into severe psychopathology, they may not fully capture the correlates of suicide risk observed operating among violence-exposed individuals living in the community. Patients hospitalized for violent injury represent a unique population characterized by elevated exposure to interpersonal violence, heightened behavioral dysregulation, and increased risk for both future violence and suicide (15, 25). Trauma centers may therefore represent a critical setting for identifying individuals at elevated suicide risk who might otherwise remain outside traditional mental health systems. Examining psychopathic traits within this context provides insight into how externalizing personality features are associated with suicide vulnerability in real-world clinical populations.

These findings suggest that the behavioral and self-regulatory components of psychopathy may be more proximal to suicide risk in violently injured adults than affective detachment or interpersonal manipulation. One possible interpretation is that impulsive, lifestyle-related traits account for part of the observed association between broader psychopathic tendencies and suicide risk, although longitudinal research is needed to evaluate this possibility. Consistent with theoretical models of violence exposure, repeated exposure to interpersonal harm may be associated with greater engagement in risk-taking behavior and altered perceptions of self-injury, thereby amplifying the impact of lifestyle-related traits on suicidal behavior (4, 26).

### Clinical implications

The present findings have important implications for suicide risk assessment in violence-exposed populations. Screening for behavioral dysregulation and impulsivity may enhance identification of individuals at elevated suicide risk following violent injury. Traditional suicide assessments often emphasize internalizing symptoms such as depression and hopelessness; however, the current findings suggest that externalizing personality traits, particularly those reflecting lifestyle instability and behavioral disinhibition, may represent an additional correlate to suicide vulnerability. Incorporating assessment of externalizing traits into trauma center or post-injury care settings may improve early identification of high-risk individuals and support more targeted intervention strategies.

### Limitations

Several limitations warrant consideration. First, suicide risk was assessed using self-report measures, which may be subject to reporting biases. Future research should incorporate multimethod assessments, including structured clinical interviews and medical record data, to strengthen measurement validity. Second, depressive symptoms were not included in the present study despite their well-established association with suicidal thoughts and behaviors. Consequently, some observed associations may partially reflect unmeasured psychiatric factors. Future research should incorporate a comprehensive assessment of depressive symptoms to clarify the unique association between psychopathic traits and suicide vulnerability. Nevertheless, the inclusion of PTSD and substance use as covariates represents an important step toward accounting for clinically relevant psychiatric correlates of suicidality and strengthens confidence in the observed associations. Third, intercorrelations among psychopathy facets may complicate the interpretation of unique facet-level regression coefficients. In particular, the reversal of the Antisocial facet coefficient when entered simultaneously with the remaining psychopathy facets may reflect shared variance and suppression-related effects rather than a meaningful inverse association with suicide risk. Accordingly, unique facet effects should be interpreted cautiously. Fourth, although the sample was demographically diverse, participants were limited to hospitalized, violently injured adults, which may limit generalizability to other violence-exposed or community populations. Fifth, the present sample was predominantly male (72%), which may have reduced sensitivity for detecting sex-specific effects. The absence of significant moderation effects should not be interpreted as definitive evidence that associations between psychopathy and suicide risk do not vary by sex. Future studies with more balanced samples are needed to further evaluate potential sex differences. Sixth, although psychopathy demonstrated significant associations with suicide risk, the proportion of variance explained by the models was modest, suggesting that lifestyle-related psychopathic traits are unlikely to function as a standalone screening tool and should be considered alongside broader clinical and contextual risk factors. At the same time, the present findings suggest that lifestyle-related traits reflecting behavioral dysregulation may represent one clinically relevant component of suicide vulnerability within violence-exposed populations and may provide useful information when considered alongside established risk factors. Finally, the cross-sectional design precludes any causal inference. Longitudinal research is needed to determine whether lifestyle-related psychopathic traits prospectively predict suicidal behavior in this population.

## Conclusions

The present study extends existing research on psychopathy and suicidality among violently injured adults. The findings suggest that the association between psychopathy and suicide vulnerability in violence-exposed populations may be more strongly related to traits reflecting behavioral dysregulation and impulsivity than to other psychopathy domains. Lifestyle-related psychopathic traits may be associated with suicide vulnerability through tendencies toward impulsive behavior and increased engagement in risk-taking during periods of distress. Future longitudinal work is needed to determine whether these processes function as mechanisms underlying the observed associations. Results highlight the importance of examining psychopathy as a multidimensional construct and suggest that externalizing personality traits represent one potential pathway associated with suicide risk among violently injured adults.

## Data Availability

The raw data supporting the conclusions of this article will be made available by the authors, without undue reservation.
